# The chemical signatures underlying host plant discrimination by aphids

**DOI:** 10.1038/s41598-017-07729-0

**Published:** 2017-08-17

**Authors:** David P. Hopkins, Duncan D. Cameron, Roger K. Butlin

**Affiliations:** Department of Animal and Plant Sciences, The University of Sheffield, Sheffield, S10 2TN UK

## Abstract

The diversity of phytophagous insects is largely attributable to speciation involving shifts between host plants. These shifts are mediated by the close interaction between insects and plant metabolites. However, there has been limited progress in understanding the chemical signatures that underlie host preferences. We use the pea aphid (*Acyrthosiphon pisum*) to address this problem. Host-associated races of pea aphid discriminate between plant species in race-specific ways. We combined metabolomic profiling of multiple plant species with behavioural tests on two *A*. *pisum* races, to identify metabolites that explain variation in either acceptance or discrimination. Candidate compounds were identified using tandem mass spectrometry. Our results reveal a small number of compounds that explain a large proportion of variation in the differential acceptability of plants to *A*. *pisum* races. Two of these were identified as L-phenylalanine and L-tyrosine but it may be that metabolically-related compounds directly influence insect behaviour. The compounds implicated in differential acceptability were not related to the set correlated with general acceptability of plants to aphids, regardless of host race. Small changes in response to common metabolites may underlie host shifts. This study opens new opportunities for understanding the mechanistic basis of host discrimination and host shifts in insects.

## Introduction

Phytophagous insects are extremely diverse and often feed on restricted ranges of host plants^1^. Co-speciation of host plants and insects is common in some taxa (e.g. fig wasps^2^), but the majority of speciation events in phytophagous insects involve shifts in their narrow host range^3^. Consequently, understanding how host shifts occur is critical in explaining a major component of biodiversity^3^. Since many phytophagous insects are serious pests of crops^4^, understanding what determines the range of host plants acceptable to an insect population also has important practical implications.

Host acceptance, host-related performance and assortative mating are often tightly inter-connected, especially for species that spend their whole lives on the host plant^5, 6^. A change in acceptance may be the first stage in a host shift, and in host-associated speciation, because it can lead automatically to assortative mating^7^. Reproductive isolation may then be reinforced by selection to increase performance on the new host^7, 8^. Therefore, understanding how host acceptance evolves in the early stages of speciation is critically important. While insects may utilise a variety of cues when making feeding decisions, chemical cues (either volatiles detected before feeding or compounds detected during feeding initiation) are very frequently involved^9^. This focuses attention on the insect chemosensory system, including chemosensory receptors, and on differences in plant chemistry among potential hosts.

Feeding stimulants and repellents have been identified in many insect-plant interactions^10, 11^ and show a wide range of chemistry^12^. These interactions are of interest in pest control but in most cases they do not explain insect specificity, i.e. why insects of closely-related species or host races accept distinct ranges of host species. Cases where a compound, or mixture of compounds, has been shown to be attractive or to stimulate feeding for one insect population but have the opposite effect for a related population, are scarce. Examples include the use of volatile blends to discriminate between hosts by divergent races of *Rhagoletis pomonella* ^13^, nicotine at low concentrations stimulating only tobacco-adapted peach-potato (*Myzus persicae*) aphid feeding^14^ and the divergent chemical content of necrotic host cactus tissue that is associated with the genetic divergence between *Drosophila mojavensis* populations^15^.

Pea aphid, *Acyrthosiphon pisum*, host races provide an excellent model to study speciation^16^ and chemically-induced host-plant discrimination. In pea aphids, host acceptance occurs when aphid stylets penetrate plant epidermal layers suggesting that interactions with compounds within plant leaves are important^17, 18^. However, while host chemistry seems to be important to host choice there has been limited exploration of the chemistry that underlies host discrimination by different races of *A*. *pisum*. There is evidence that divergence in genes involved in recognition via chemoreception^19^, manipulation via salivary proteins^20^ and detoxification via P450 proteins^21^ has been associated with host shifts. Whatever the mechanism for chemical recognition, it leads to performance differences and assortative mating^22^. These conditions result in reproductive isolation and genetic differentiation among races^22^.

Here, we have employed untargeted metabolomic analysis, using matrix-assisted laser desorption/ionisation time of flight (MALDI TOF) mass spectrometry of metabolites extracted from leaves, to characterise variation in potential chemical cues among host plants and related species. We chose the untargeted MALDI-TOF method because we had no strong *a priori* reasons to focus on particular metabolite classes. Given suitable controls and replication, this method is sufficiently repeatable for relative abundance of a wide range of compounds to be compared with insect behavioural responses^23^. We used leaf extracts because aphid feeding decisions are known to be influenced by cues detected as the aphid stylus passes through the apoplast and samples cell contents, before reaching the phloem^18, 24^. Host acceptance by aphids was measured using electrical penetration graphs (EPG). The EPG technique incorporates the aphid and its host plant into an electrical circuit and then records changes in electrical resistance that reveal the activity of the aphid stylus within the plant, before and during phloem feeding^25^. Information provided by EPG can be interpreted to provide counts of the instances and measure durations of specific aphid probing and feeding behaviour patterns. Four clones of the pea aphid, two that are adapted to feed on *Medicago sativa* (‘MS aphids’ from now on) and two that are adapted to *Trifolium pratense* (‘TP aphids’) were tested on 19 plant species in the genera *Medicago* and *Trifolium*. This wide range of hosts provided us with the statistical power to identify candidate metabolites capable of explaining aphid discrimination from amongst the large number of compounds in the metabolomic profiles.

Acceptance was summarised for each combination of host species and aphid races as either the time spent in the E2 phase, a measure of sap ingestion during phloem contact (‘E2 profile’), or as the first linear discriminant axis (‘LD1 profile’) based on 60 variables extracted from EPG traces. While phloem ingestion may not represent the specific point of host acceptance, E2 profile was used in this study because feeding is the clearest behavioural indicator of positive host response. This measure was complemented by the LD1 profile, which provided a broad summary of the aphid probing and feeding behaviours. In either case, we derived scores for ‘discrimination’ by MS vs TP aphids and for ‘overall acceptability’ by all aphids. The relationships of these scores to aphid performance were tested. Random forest (RF) regression was then used to search the polar and non-polar fractions of the metabolomics data for the best predictors of discrimination or acceptability.

## Methods

### Aphid culture

Four asexually-maintained lineages (clones) of *A*. *pisum* were used: the *Medicago sativa* specialised clones LSR1^26^ and L9Ms_052 (source SE France, supplied by JC Simon, INRA, Rennes), and *Trifolium pratense* specialised clones YR2^27^ and L7Tp_232 (source SE France, supplied by JC Simon, INRA, Rennes). Aphids were kept at a density of 10–15 individuals per 10 day old bean, *Vicia faba*, plant (variety ‘The Sutton’). This host is accepted by aphids of both races and so provides a common environment, controlling aphid condition and experience. Age-controlled aphids were produced by exposing plants to adult aphids for 24 hours then removing adults. Progeny were then left to develop for 14 days. On the day of use, aphids were taken from *V*. *faba* plants and starved for 1 hour before experimentation.

### Plant culture

In total, 19 species of plant were used from the genera Medicago (*M. arabica, M. orbicularis, M. littoralis, M. tornata, M. turbinata, M. laciniata, M. lupulina, M. truncatula, M. sativa*) and *Trifolium* (*T. ambiguum, T. striatum, T. nigrescens, T. repens, T. pratense, T. ochroleucum, T. rubens, T. semipilosum, T. dubium, T. pallidum*) (see Supplementary Table S1 for seed sources). Seeds were sterilised by soaking in saturated calcium hypochlorite solution for 2 minutes and then plated out in Petri-dishes containing 1.2% plant agar containing 50 mg/ml gibberellin (source Sigma-Aldrich UK^©^). They were left to germinate for one week at 20°C day and 15°C night temperatures, with 16 hour day length. Resulting seedlings were transferred into seed trays containing 4:1 sand and John Innes no.2 compost mix, covered with a lid for the first 14 days to retain humidity. Plants were grown for five weeks in total and watered twice weekly with distilled water. Plants were fed twice with Rorison’s solution, in the 4^th^ week with 40%, and in the 5^th^ week with 20% of full strength solution.

### Measuring aphid host preference using EPG

Aphid acceptance was measured by the electrical penetration graph (EPG) method^25^ using a DC Giga-8 sourced from EPG systems (www.epgsystems.eu). The EPG technique records the changes in potential difference as the resistance to a weak electrical current is affected by the progression of an aphid stylet through a leaf to the phloem^25^. Changes in potential difference can be interpreted as “waveforms” that represent particular aphid behaviour patterns^24^. Behaviour for each aphid was recorded for 6 hours. Eight EPG recordings were performed per day. A blocked design was used, with each plant species represented during each five week block, and on each week-day within a block. Plant species–aphid clone combinations were set up in duplicate within days so that occasional recording failure (e.g. due to the aphid leaving the test plant) did not interfere with the block design. Five to 11 aphids were recorded successfully for each plant species and aphid clone combination.

### EPG profile of acceptance

EPG traces were interpreted using the Stylet + software (www.epgsystems.eu) and the waveform key in Sarria *et al*.^28^. Annotated EPG recordings were then entered into the MicroSoft Excel macro “workbook for EPG parameter calculations of EPG data: version 4.4”^28^ to calculate 119 separate behavioural measurements for each recording. Missing values were imputed using the RFimpute() function in R^29^. Uninformative variables were cleaned from the data set by removing waveforms with 50% or more values equal to zero and then removing one variable from any pair of variables with correlation >0.80. After this 60 variables remained (Supplementary Table S2). We tested the impact of these data manipulation steps on subsequent analyses (Supplementary Fig. S1). Two summary statistics were generated for each recording. The first E2 profile was the “Total duration of E2 waveform”, which represents the time an aphid passively ingests phloem sap^30^. The second statistic, LD1 profile, was the score on the first axis of a linear discriminant analysis of all 60 EPG waveforms, calculated for each aphid race separately, with plant species as the grouping factor. This first LD axis explained 24.5% and 22.2% of among plant variance in EPG scores for *Medicago* adapted aphids and *Trifolium* adapted aphids, respectively.

The difference between the means of the E2 or LD1 profiles for the two aphid races was calculated to provide a measure of discrimination and the sum of the means was used to measure overall acceptability of each plant species. The EPG data summaries were then compared to the data on aphid performance on plants growing under the same conditions using Pearson’s product-moment correlation coefficient across the means for all plant species. Performance was measured as the number of live young produced by a single 10 day old adult aphid (reared on *V*. *faba*, as detailed above) over a seven day period, following transfer to a fresh test plant. Due to poor germination there was insufficient plant material of *T*. *ochroleucum*, *T*. *pallidum* and *T*. *semipilosum* to use in this part of the study. For the performance measure, there were three to eight replicates for each clone-plant combination and six to 16 replicates for each race-plant combination.

### Plant metabolomic profile

Half-way through the EPG data collection period the first fully formed leaf from each of 5–7 plants of each species was cut, weighed and then quenched in liquid nitrogen. Metabolites were extracted from the frozen leaf material using the cold extraction methanol-water-chloroform method^31^. From this extraction two phases, polar and non-polar, were separated for analysis.

The concentration of each extract was adjusted according to the original leaf weight to be equal to the concentration of the smallest leaf, in order to account for a large difference in leaf sizes between species. Extracts were then diluted by 50% with methanol. Metabolic profiles were recorded using MALDI-TOF mass spectrometry (full instrument settings in Supplementary Information). This is an established high-throughput method with a large mass range (50 Da to >1000 Da) that allows for rapid and unbiased analysis of large sample numbers^23^. Briefly, 10 μl of extracts were mixed 50:50 with α-Cyano-4-hydroxycinnamic acid (α-CHCA) at a concentration of 25 mg/ml in 25% methanol 75% ethanol (v/v) as a matrix^32^ and crystallised onto a 96 spot MALDI target plate. Each extract (biological replicate) was spotted in triplicate and uniformity of crystallisation ensured by examination under a microscope. Analyte:α-CHCA spots were ionised for 60 seconds with the laser traced over each spot in a spiral pattern and the machine setup for samples determined by optimisation of matrix to analyte concentrations and laser intensity to avoid fragmentation. Internal amino acid standards were regularly applied to check for ion suppression. The spectra obtained for the triplicate samples were combined using in-house software^33^ and the match for technical replication was checked for every mass bin in the run to account for any artefact peaks. Metabolite profiles for individual plants were then generated by binning the crude m/z values into 0.2-unit bins (m/z bin) and the relative mass abundances [% total ion count (%TIC)] for each bin were summed^31^. Principal Components Analyses (PCA), using the prcomp() function in Base R (https://www.R-project.org/), were used to provide an over-view of the main axes of variation and to detect outliers. Partial Least Squares methods were used to conduct discriminant analyses (PLS-DA), in the ropls package in R^34^, in order to detect m/z bins that discriminate among plant species.

### Plant metabolome by aphid phenotype comparison

In order to test the quantitative responses of aphids to 19 plant species with abundances of many compounds detected in the metabolomics profiles, we required an efficient and powerful multivariate regression approach. Therefore, we used Random Forest regression. This is a machine-learning approach based on decision trees that has been shown to provide high prediction accuracy and reliable ranking of variable importance for biological data from high-throughput technologies^35^.

The discrimination and overall acceptability scores derived from the EPG data were used as response values to regress against the metabolic profiles of individual plants using the randomForest package in R^29^. Each random forest model was run with 1000 iterations. To account for uncertainty in the mean discrimination and acceptability EPG profile scores used as response variables, we ran each RF analysis 500 times. For each run, an EPG score for each plant species was drawn randomly from a distribution defined by the observed mean score and its standard error. We then recorded the rank value of the RF importance (measured as mean decrease in Gini) for each m/z bin. Median ranks were used to sort m/z bins and inter-quartile ranges of the ranks were used to assess consistency of variable importance. The highest ranking m/z bins were included in linear regression models, to assess the proportion of variance they explained in aphid discrimination or overall acceptability of plants.

In total, eight RF models were analysed, one for each combination of discrimination and overall acceptance, LD1 and E2 profiles, and polar and non-polar plant metabolomic data. Results were then compared across the RF models to identify m/z values with high importance held in common. For bins of high importance, we examined correlations between the behavioural scores and relative abundance in m/z bins.

To test the RF model stability, 500 jack-knifed versions of the EPG data set were created in which one value was removed, at random, from each of the plant-aphid combinations before calculating the means. The RF analysis was run for each data set and the median rank importance and the interquartile ranges were calculated for each m/z value. We also repeated the RF analyses after exclusion of outliers detected in the PCA on metabolomics data. We present the analyses using the full data set unless otherwise stated.

### Characterisation of significant m/z (masses)

The putative identities of compounds in m/z bins with high importance in the RF analyses, or in pathways connected to these compounds, were investigated using the comprehensive Kyoto Encyclopedia of Genes and Genomes (KEGG) (www.genotome.jp/kegg) and MetaCyc Compound (www.biocyc.org) databases. In order to assign an identity to a given metabolite (m/z) the Metabolomics Standards Initiative recommends a minimum of “two independent and orthogonal data relative to an authentic compound analyzed under identical experimental conditions to validate non-novel metabolite identifications” and considers the combination of accurate mass and tandem MS as an acceptable coupling of methods representing best practice^36, 37^. Therefore, to confirm the identity of the putatively identified compounds based on accurate masses, we obtained standard chemicals (sourced from Sigma-Aldrich UK^©^) which were then analysed in ESI TOF tandem mass spectrometry (tandem MS) to generate specific fragmentation patterns. The appropriate m/z accurate masses corresponding to our putative identifications were also fragmented by tandem MS and were compared to fragmentation patterns generated from the appropriate standard (full instrument settings in SI). Finally, the KEGG pathway database (www.genome.jp/kegg/pathway) was used to investigate the relationships between the different putative compounds in plant metabolism. Where standards were not available, *in silico* models of the likely fragmentation patterns were generated using ChemDraw std. ver. 13.0, Perkin Elmer.

### Data accessibility

EPG, performance and metabolomics data: Dryad doi:10.5061/dryad.h4p2b.

## Results

### EPG profiles

**Figure 1:**
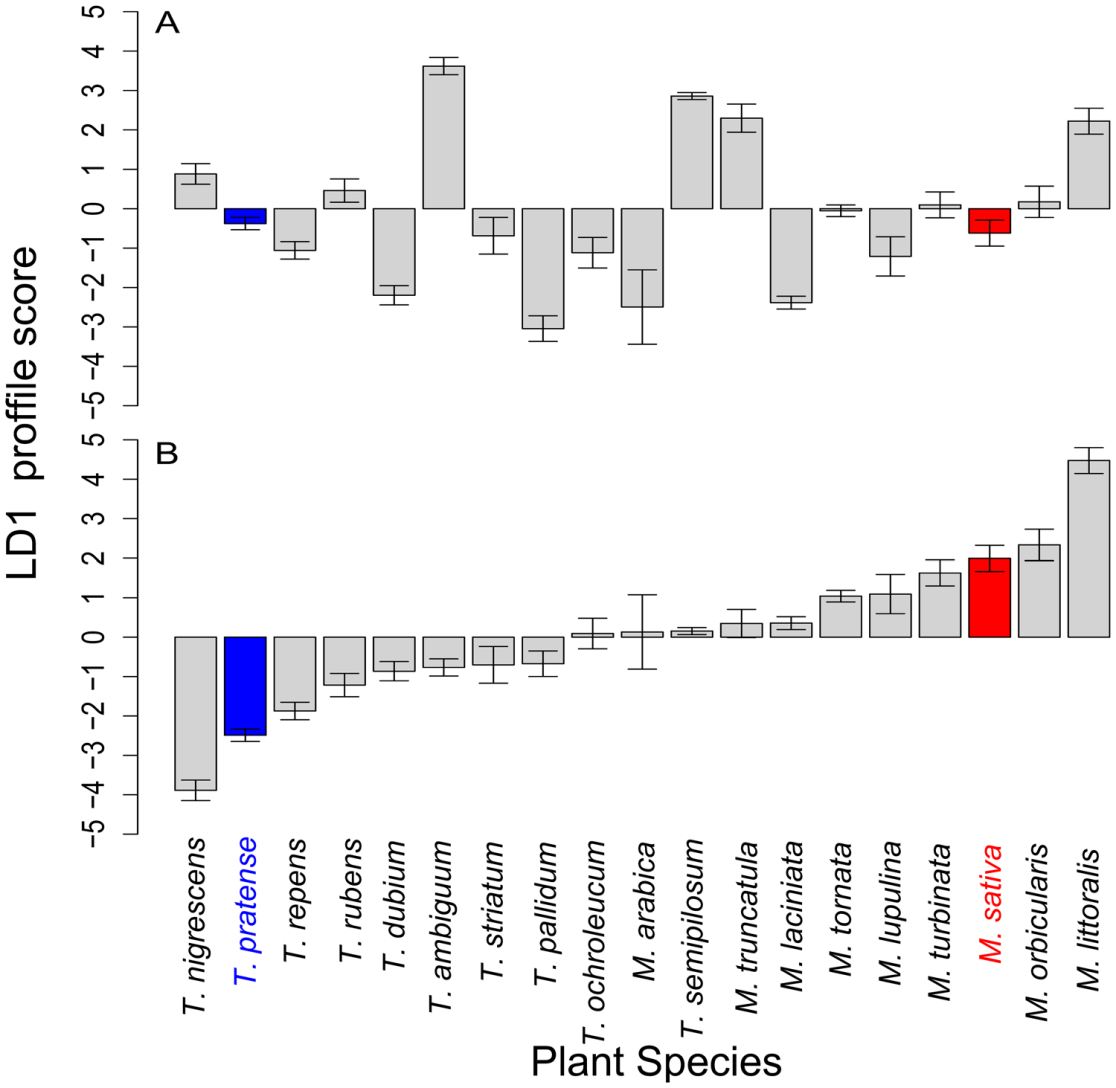
EPG profiles for MS and TP aphids on each plant species. (**A**) Overall acceptance profile with positive LD1 values indicating preference for, and negative values indicating rejection of plants. (**B**) Discrimination profile with positive values indicating greater acceptance by MS aphids and negative values greater acceptance by TP aphids. Red = MS aphid native host, blue = TP aphid native host. There were 2 clones per race, 5–11 replicates per clone and plant species. Mean ± SEM.

Both LD1 and E2 profiles from the EPG showed a continuum in overall acceptability and discrimination across the 19 host plant species (Fig. 1, Supplementary Fig. S2) and these measures were uncorrelated (E2 r = 0.18, *P* = 0.46; LD1 r = 0.13, *P* = 0.58). The LD1 and E2 profiles were strongly correlated (MS aphids r = 0.92, *P* < 0.001; TP aphids r = 0.92, *P* < 0.001) and aphids of different clones, within races, showed very similar profiles (LD1: MS aphid clones r = 0.88, *P* < 0.001; TP aphid clones r = 0.75, *P* < 0.001). Note that aphid discrimination among plants showed a clear pattern separating *Medicago* from *Trifolium* species (Fig. 1b). There were significant correlations between aphid performance variation across plant species, measured as fecundity of single adults over seven days, and acceptability measured as either E2 (MS aphids r = 0.76, *P* < 0.001; TP aphids r = 0.79, *P* < 0.001) or LD1 profile (MS aphids r = 0.80, *P* < 0.001, TP aphids r = 0.76, *P* < 0.001) (Supplementary Fig. S2). This is in line with previous observations that EPG provides meaningful measures of host acceptance^18, 38, 39^.

### Metabolomics and identification of candidate masses

Unsupervised principal component analysis of metabolomic profiles of plants revealed considerable overlap among host plant species in composition of both polar and non-polar fractions and only weak separation between the plant genera (Supplementary Fig. S3). We detected a single outlying individual of *T*. *dubium* in the non-polar data set and a single outlying individual of *M*. *littoralis* in the polar data set. The PLS-DA by plant species provided better separation of taxa, as expected (Supplementary Fig. S4). Loadings on the major discriminatory axes did not reveal any m/z bins that contributed particularly strongly to this separation. Overall, the weak discrimination among plant species indicates that any chemical cues that aphids might respond to represent only a small proportion of total variation in the metabolome.

**Figure 2:**
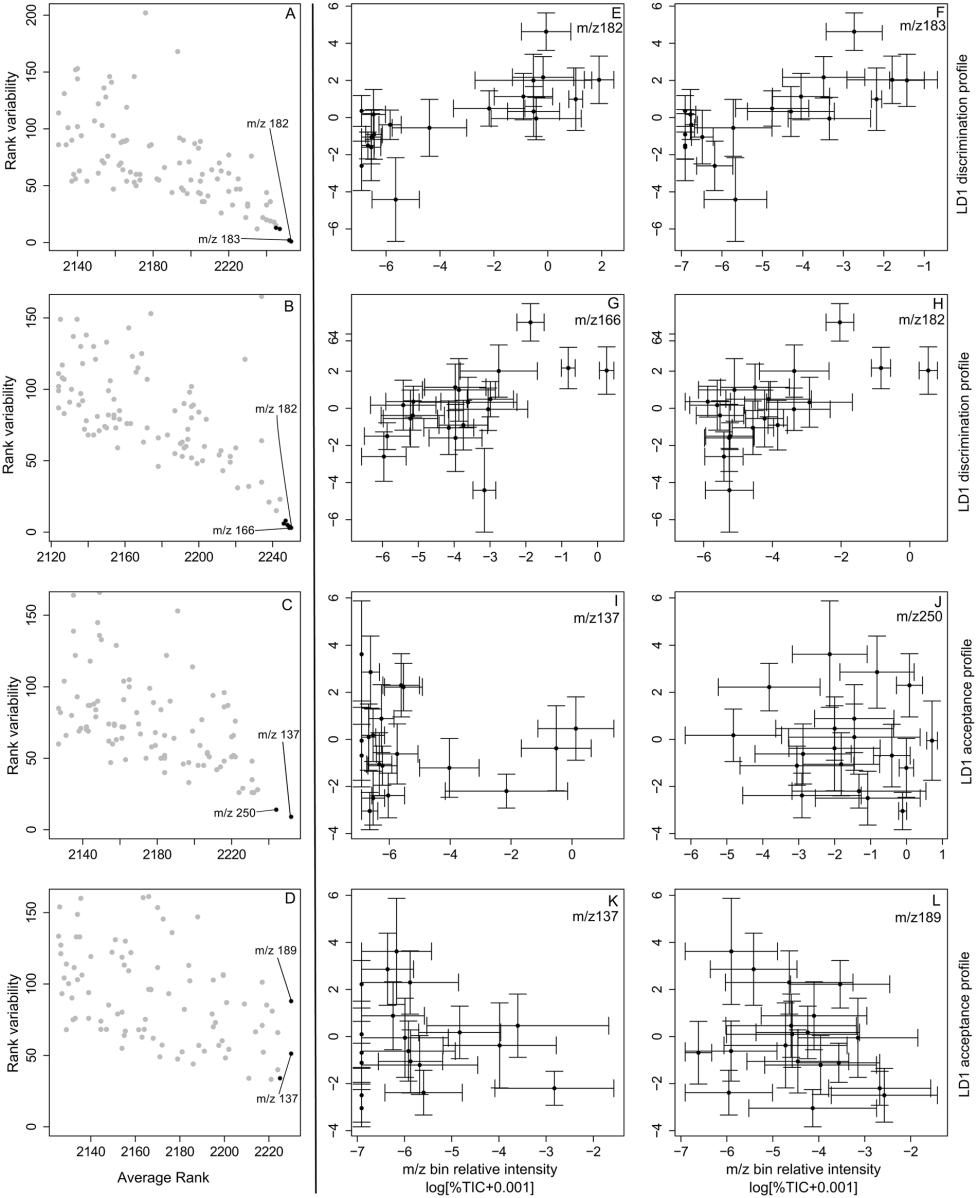
Results of Random Forest analyses and correlation of abundance in top ranking m/z bins with LD1 profile. A-D; Rank variability (interquartile range) as a function of median rank for the RF importance score (mean decrease in Gini coefficient) of the top 100 m/z bins from 500 runs of the RF regression models: LD1 discrimination profile against (**A**) polar and (**B**) non-polar metabolic data and LD1 overall acceptance profile against (**C**) polar and (**D**) non-polar metabolic data. m/z bins with the highest average rank best explain variation in LD1 profile and greater confidence can be placed in bins with low rank variability. Points in black are key m/z bins used for further investigation. (**E**–**L**) Correlations between key m/z bin values highlighted by RF models and LD1 scores. Mean ± SEM, Significance tested using Spearman’s rank correlation with FDR correction: NS – not significant, *P < 0.05, **P < 0.001, ***P < 0.001.

In order to unpick this complexity, we applied a Random Forest (RF) approach to resolve whether any metabolites could explain the variance in aphid behaviour. RF regression for discrimination profile, using relative abundance, expressed as % total ion count in either 955 polar or 965 non-polar mass/charge (m/z) bins (representing individual compounds or small groups of compounds), identified a small number of bins with consistently high explanatory power, as indicated by importance rank (Fig. 2A,B). The 8 top scoring m/z bins, from the polar and non-polar data sets together explained, 44% of the variation in the LD1-based discrimination score. Similar results were obtained when using E2 discrimination scores (Supplementary Fig. S5) and the analysis also confirmed that m/z values with the highest importance also had the highest stability (Supplementary Fig. S6). In contrast, RF regression for the LD1-based overall acceptance score revealed no specific masses with consistently high explanatory power (Fig. 2C,D) and the top 8 m/z bins together explained less than 1% of variation.

Comparison of RF models of aphid discrimination with those for overall acceptance, showed that very different combinations of m/z bins were implicated (Supplementary Table S3). Analyses based on E2 and LD1 discrimination profiles gave similar results (Supplementary Table S3). RF models were robust to the removal of outlier plant individuals, especially for the LD1 discrimination score (Supplementary Table S4). This suggests that a small number of metabolites contribute to the distinct chemical signatures that underlie plant discrimination by aphids of different race and that the compounds involved are different from those that explain overall acceptability. Seven top scoring m/z bins for aphid discrimination, from both extracts, were considered for further analysis (Table 1). Of these, m/z bins 182 and 166 in both polar and non-polar plant extracts and 183 in the polar plant extracts were at high concentration in plants associated with high acceptance scores by MS aphids and low acceptance scores by TP aphids (Fig. 2E–H). Compounds in these bins were generally more abundant in *Medicago* than in *Trifolium* species. In contrast, m/z bins 269, 291, 292 and 285 in the non-polar extracts were at high concentration in plants associated with acceptance by TP aphids and rejection by MS aphids (Supplementary Fig. S7). Compounds in these bins were abundant in *T*. *pratense* and *T*. *nigrescens*, in particular. Searches of online metabolome databases (www.genome.jp/kegg/pathway, www.biocyc.org) revealed putative identities for m/z bin 166 as L-phenylalanine and m/z bin 182 as L-tyrosine (in addition, since polar m/z bin 183 differs by only one proton from m/z 182 this may also be L-tyrosine). Comparison of tandem mass spectrometry fragmentation patterns of these bins to standards, confirmed these putative compound identities (Supplementary Fig. S8). Identification of other m/z bins has remained inconclusive as tandem MS fragmentation patterns failed to match fragmentation patterns of standards for candidate compounds (Supplementary Fig. S8).

**Table 1:** Mass/charge (m/z) bins from polar and non-polar samples identified by RF models.

Polar			Non polar		
m/z bin value	ρ	*P*	m/z bin value	ρ	*P*
182	0.76	>**0**.**001**	182	0.70	**0**.**001**
183	0.73	>**0**.**001**	166	0.65	**0**.**003**
166	0.79	>**0**.**001**	269	−0.53	**0**.**018**
			285	−0.53	**0**.**019**
			291	−0.18	0. 46
			292	−0.32	0.18

The relationships between abundance (% TIC per bin) and the LD1 discrimination profile were tested with Spearman’s rank correlation (S). DF = 17.

### L-phenylalanine and L-tyrosine pathway analysis

**Figure 3:**
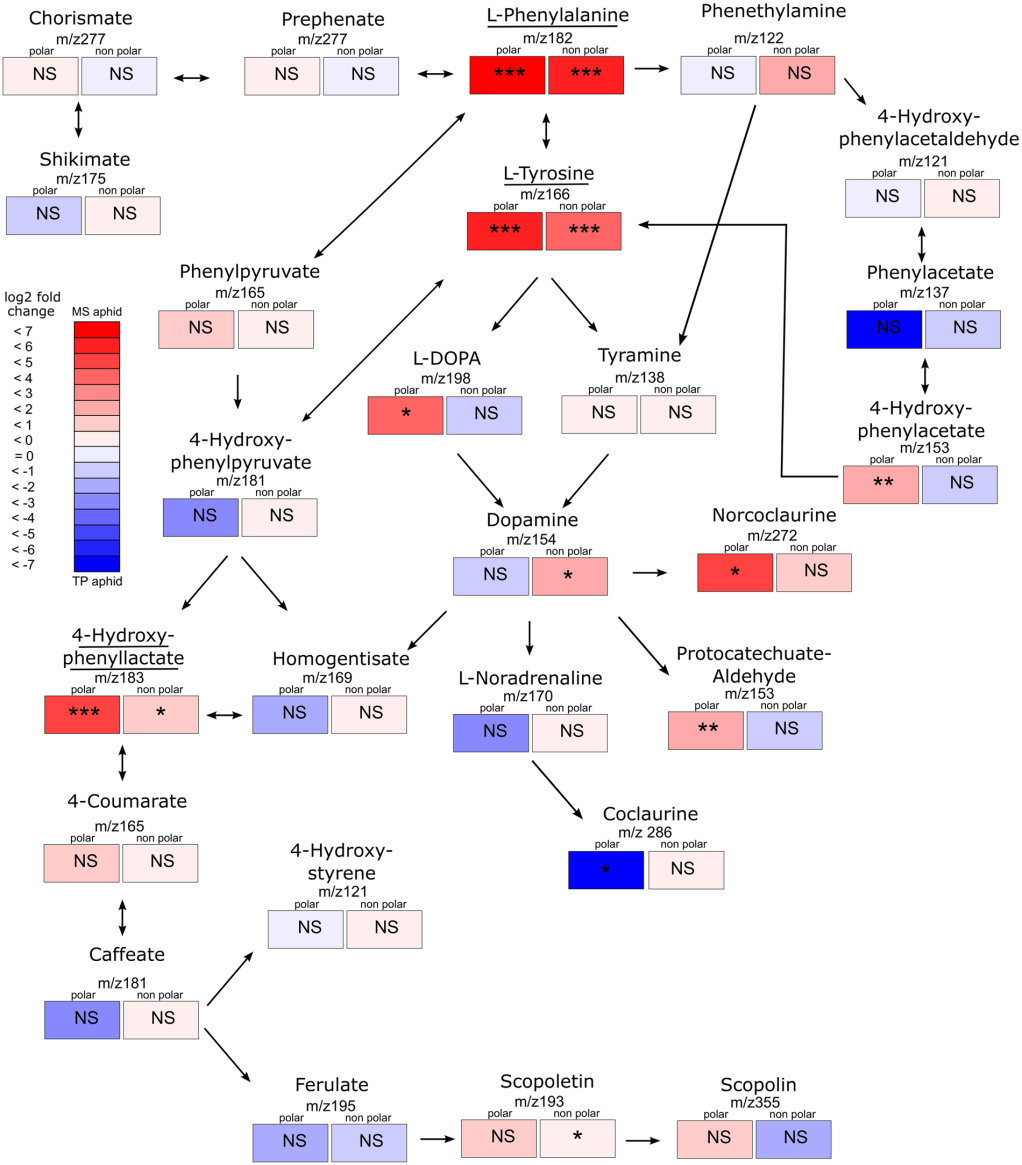
Plant metabolic pathways stemming from L-phenylalanine and L-tyrosine. Colour represents the abundance difference between the three most divergent *Medicago* (*M*. *sativa* (n = 6), *M*. *littoralis* (n = 6), *M*. *orbicularis* (n = 6)) and *Trifolium* species (*T*.*pratense* (n = 5), *T*. *nigrescens* (n = 6), *T*. *repens* (n = 7)) for the discrimination LD1 score (log(fold change) in the mean % TIC of the m/z bin putatively associated with the compound). The pathway is based on the *M*. *truncatula* pathway in the KEGG database (www.genome.jp/kegg/pathway.html). Significance was tested by comparing the difference in the combined log %TIC between the *Medicago* and *Trifolium* groups using a linear regression model (using lm() function in R). Compounds underlined are those with putative m/z bins identified in RF models for both discrimination and overall acceptance.

Pathway analysis by accurate mass identification (to three decimal places) of downstream compounds in the metabolic fingerprint associated with L-phenylalanine and L-tyrosine showed a number of putative compounds that had a greater abundance in plants with higher MS aphid acceptance (especially *M*. *littoralis*, *M*. *sativa*, *M*. *orbicularis*) than in plants accepted readily by TP aphids (*T*. *nigrescens*, *T*. *pratense* and *T*. *repense*) (Fig. 3). These included m/z bin 198 (polar: F = 4.97, df = 1, 34, *P* = 0.033) and m/z bin 154 (nonpolar: F = 5.34, df = 1, 31, *P* = 0.028), putatively identified as L-DOPA and dopamine, respectively. Other compounds downstream from dopamine may also be associated with discrimination by aphids. Bins putatively identified as 4-hydroxyphenyllactate (m/z 183 in polar samples: F = 33.83, df = 1, 34, *P* = 0.001, nonpolar samples: F = 4.6, df = 1,31, *P* = 0.04), norcoclaurine (m/z 272 in polar sample: F = 3.08, df = 1, 31, *P* = 0.046) and 4-hydroxyphenylacetate (m/z 153 in polar sample: F = 8.47, df = 1, 34, *P* = 0.006) all correlated with LD1 discrimination score. Only one m/z bin (non-polar 286) was consistently at higher concentrations (F = 6.20, df = 1, 31, *P* = 0.018) in plants preferentially accepted by TP aphids; putatively identified as a downstream metabolite in this pathway (coclaurine). Overall, plants preferentially accepted by MS aphids had elevated activity in L-phenylalanine and L-tyrosine associated metabolic pathways and multiple compounds in these pathways were correlated with differential aphid acceptance.

## Discussion

Our strategy of analysing the differential responses of aphid host races to 19 different plant species, in relation to untargeted metabolomic profiles, revealed a very small number of m/z bins capable of explaining a high proportion of the variance in aphid discrimination, i.e. the relative acceptability of the plants to aphids from different host races. The compounds that explained variation in discrimination by host races were different from the more complex set underlying general acceptability to aphids. In plants accepted by MS aphids and rejected by TP aphids, m/z bins 182 and 166 (both fractions) had consistently high values and these bins have been identified with high confidence as L-phenylalanine and L-tyrosine. Our data suggest that plant discrimination by aphids occurs because of either a direct response to these metabolites or a response to metabolites with correlated abundance, possibly in associated pathways that were found to be upregulated.

There is evidence to suggest that aphid discrimination of host plants is due to an interaction with plant chemistry early in plant penetration, including contact with cell contents before the stylus reaches the phloem^17, 18, 24^. Our EPG approach focused attention on this early phase of the interaction and this may partly explain why we have identified a potential role for primary metabolites in discrimination, rather than the plant secondary metabolites commonly associated with resistance to aphids. Secondary metabolites are often induced in response to feeding^12^ and so may be less likely to play a role in the earliest phase of discrimination. Constitutive deterrent compounds may have more of a role in determining overall acceptability, which we have shown to be uncorrelated with discrimination. Nevertheless, we do show that, within aphid races, the short-term behavioural response measured with EPG is strongly correlated with performance, a longer-term measure of plant suitability for aphid feeding. This suggests that the signals detected during probing or initial feeding are good predictors of later interactions. For the natural hosts of the two aphid races examined, this is expected from co-evolutionary interactions^12^. However, it is also true here for multiple plant species that are not natural hosts for either aphid race, with rankings differing between races. This is more readily understood if the metabolites that we have identified, or metabolites with closely correlated variation among plant species, are directly implicated as nutrients or antifeedants of specific relevance to the differently-adapted races. In this context, it is noteworthy that aphid discrimination behaviour distinguishes between the plant genera, whereas neither overall acceptance nor general metabolic profile shows this pattern. This further suggests that a subset of metabolites implicated in aphid discrimination varies among plant species differently from the majority of metabolites.

Variation in nutritional requirements among *A*. *pisum* clones has been observed previously^40^: one of six clones tested lacked the ability to biosynthesise the amino acid arginine due to a specific mutation in its *Buchnera* symbiont. Therefore, it is plausible that the amino acids identified here might differ in nutritional value among clones of different aphid races. Previous studies have also shown that L-tyrosine can act as an allelochemical, deterring insect herbivory, although at concentrations that are probably much higher than observed here^41, 42^. However, a role in discrimination, i.e. differential acceptability by closely related species or races, has not been suggested previously. Alternatively, metabolism of tyrosine may generate active compounds or other defence signalling pathways. The identification of both L-phenylalanine and L-tyrosine in this study is of particular interest as they are directly linked by the same plant metabolic pathway (Fig. 3). The presence of these two compounds suggests that aphid discrimination may respond to expression of this pathway, or to metabolites produced by this pathway (Fig. 3), or possibly to larger compounds for which these metabolites are break-down products. Indeed, tyrosine metabolism is the precursor of a number of biologically active compound families, including several that have been implicated in aphid feeding. For example, aphid feeding may result in increased tyrosine decarboxylase expression^43^ leading to production of defensive alkaloids or structural compounds strengthening cells walls^44^. Dopamine and L-DOPA are important secondary metabolites in insects used in the production of melanin, necessary for cuticle formation and for insect cognition, including the regulation of feeding behaviours^45, 46^. Metabolism of L-tyrosine to L-DOPA and dopamine leads to a host of interesting plant alkaloids that could function as aphid deterrents, including the ipecac alkaloids, isoquinoline alkaloids, glucosinolates and isoflavonoid, benzylisoquinolines, morphine, norepinephrine, epinephrine and phenethylamine^47–50^. Indeed both epinephrine and norepinephrine have previously been identified in bean (*Phaseolus vulgaris*) and pea (*Pisum sativum*) plant material^48^. Both L-tyrosine and L-phenylalanine are precursors in the pathways leading to phenylpropanoids, flavonoid and isoflavonoid, compounds that could potentially influence host acceptability^48, 50, 51^. All of the above compounds can be biologically active at very low concentrations, can be readily modified to other compounds and are often produced predominantly within the leaf^48^. Critically, none of these previous studies has considered the role of these compounds in discrimination, as opposed to overall acceptability: two types of response that are uncorrelated in our data.

Our results reveal strong correlations between aphid discrimination and a small number of metabolites: L-tyrosine, L-phenylalanine and a few additional m/z bins for which positive identifications have not yet been possible. Further work will be needed to confirm the identity of these compounds and to test the causative roles of these compounds or of metabolically-related compounds. We considered nearly 2000 m/z bins in our analysis but this is by no means the whole metabolome of the leaves. There are limitations with untargeted MS techniques using MALDI. One is ion suppression (or competitive ionization)^52^, which occurs when MS is used to test a matrix of multiple compounds: ionisation can favour compounds at higher concentrations or with particular chemistries, such as amino acids, which then bias the relative abundance estimate^53^. This increases the likelihood of false negatives in the RF model (i.e. missing the importance of compounds at lower concentration or unlikely to accept positive charges). Additionally, relative abundance estimates are subject to error: this may also obscure any relationship with insect response but cannot explain the strong associations seen in our data across multiple individuals of 19 plant species. Finally, confident identification of m/z bins is not always possible as i) some compounds overlap in their spectra and cannot be separated by tandem MS, ii) standards of putative compounds are not available for tandem MS, iii) m/z values in online databases vary with techniques and machinery and iv) our knowledge of the metabolomes of plants is incomplete so novel chemistry may not be easily identified. These are common challenges shared across the field of metabolomics^53^. However, using untargeted metabolic profiling has some major advantages. Most importantly it avoids bias from any preconceived expectations. This allows one to discover new compounds that would otherwise not be discovered with targeted approaches. Crucially, our use of many plant species provided the statistical power to enable important signals to be detected. Targeted approaches can now be used to follow up our discoveries.

Our observation that a small number of plant compounds can explain nearly half of the variance in discrimination by aphids is compatible with recent work on the genetic basis of host race formation in *A*. *pisum*. Chemoreceptors (CR) could play an important role in aphid-plant co-evolution, with CR gene families having undergone significant and recent expansions, partly driven by positive selection^54^. Smadja *et al*.^19^ found that only a few members of the CR gene families were more genetically divergent between host races than expected under neutrality, perhaps corresponding to the small number of compounds identified here. In addition to sequence divergence, copy number variation (CNV) between aphid races has been documented for both CR and cytochrome P450 genes^21^. CNV between races was particularly strong for gustatory receptor loci, suggesting that copy number evolution may be important in specialisation, perhaps through effects on gene expression^21^. The divergent evolution of a subset of odorant and gustatory receptors, points to a mechanism of host plant specialisation based on aphid perception of plant chemical constitution^9^. On the other hand, the divergent copy number of P450 genes suggests divergent adaptation of aphid ability to metabolise plant allelochemicals^21^. In support of this idea, aphids are well known for their ability to both avoid and to suppress plant chemical defences^55, 56^.

Our study adds significantly to the evidence that aphid host race formation and speciation is driven by specialised adaptations to the chemistry of plants via the perception of a few specific plant compounds. While previous work has suggested that *A*. *pisum* interactions with leaf chemistry in early plant probing are key to their host acceptance, until now little progress has been made in identifying the plant chemistry responsible. Our results provide an important step forward in identifying critical plant metabolites involved in divergent host selection, using a novel approach that can readily be applied in other systems.

## Electronic supplementary material

Supplementary Information
