# Supplementary material for: The chemical signatures underlying host plant discrimination by aphids
Source: Sci Rep. 2017 Aug 17;7:8498. doi: 10.1038/s41598-017-07729-0 (PMC5561273; doi:10.1038/s41598-017-07729-0)
Supplement: Supplementary file 1 — Supplementary Information [file 41598_2017_7729_MOESM1_ESM.pdf]

The chemical signatures underlying host plant discrimination by aphids

David P. Hopkins, Duncan D. Cameron and Roger K. Butlin*
Department of Animal and Plant Sciences, The University of Sheffield, Sheffield S10 2TN, UK
*Corresponding author: r.k.butlin@sheffield.ac.uk, Tel. +44 (0)114 2220097

Supplementary information

Aphid clone information

Four asexually-maintained lineages (clones) of *A. pisum* were used: the *Medicago sativa* specialised clones LSR1[24] and L9Ms_052 (source SE France, supplied by JC Simon, INRA, Rennes), and *Trifolium pratense* specialised clones YR2[25] and L7Tp_232 (source SE France, supplied by JC Simon, INRA, Rennes).

Mass spectrometry settings:

MALDI TOF

Optimisation tests found the greatest number of counts of CHCA parent ion fragments was recorded by a using target intensity of 200 and step rate of 30.

Machine name: Waters Synapt G2

Scan conditions
Polarity = positive
Mode = Set to sensitivity mode
Scan rate = 1 scan per second
Scan duration = 120 seconds
Step rate = 50
Laser energy = 200

Voltage settings
Sample plate = 0
Extraction -= +10
Hexapole = 11
Aperture = 7

Tandem MS

Machine name: ABI Sciex Qstar Elite

Scan settings
Scan type = product ion
Polarity = positive
Mass range = m/z 50 to m/z190
Scan length = 5 minutes
Cycles = 300
Accumulation time = 1 scan per second

Syringe pump method
Diameter = 2.3mm
Flow rate = 10.0 µL/min

Compound
Declustering Potential= 45.0
FP = 265.0
DP2 = 15.0
CE =30.0
CAD = 4
IRD = 6.0
IRW = 5.0

Source/ Gas
Ion source Gas 1 (Gs1) = 27.0
Ion source Gas 1 (Gs1) = 0.0
Curtain Gas (CUR) = 20.0
Ion spray voltage (IS) = 3500.0
Temperature (TEM) =0.0
Accumulation time = 0.999942 (sec)

Resolution
Ion energy (IE1) = 1.0
Focusing lens (IQ2) = 8.5
Collision cell rod offset (RO2) = 8.5
DC Quad lens horizontal focus (GR) = 7.8
DC Quad lens vertical focus = (TFO) = 9.8
DC Quad lens steering (TST) = -0.5

Detection
Detector (CEM) = 2500.0

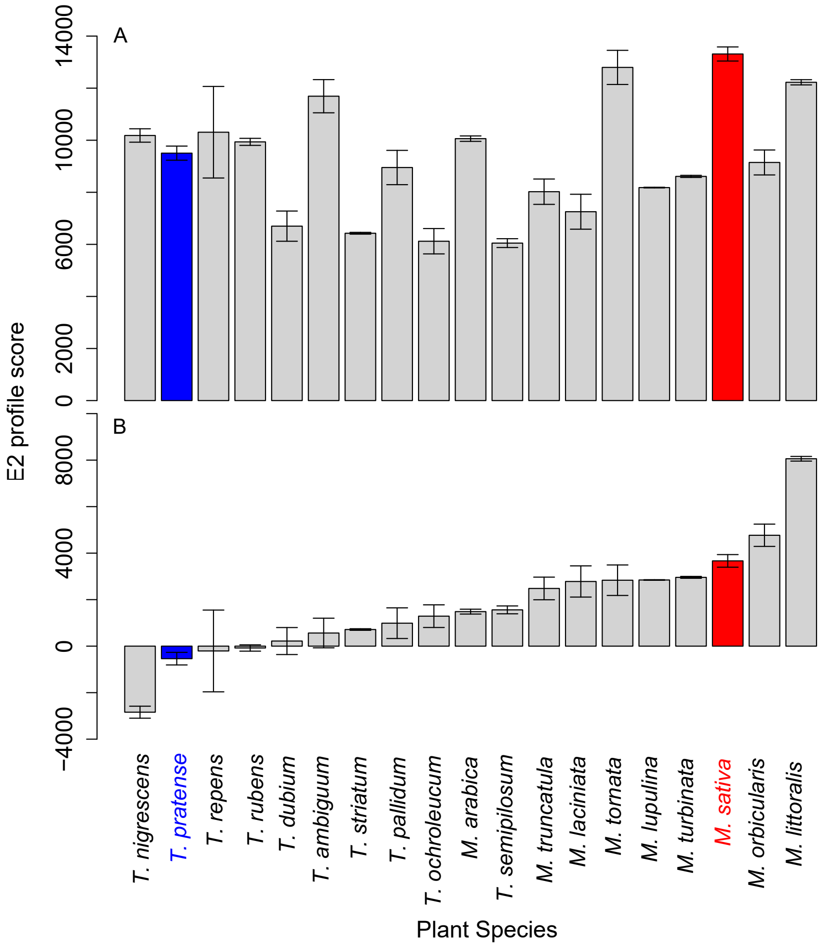

Figure S1. EPG E2 profiles for MS and TP aphids on each plant species. (A) Overall acceptance profile. (B) Discrimination profile with positive values indicating greater MS aphid acceptance, and negative values greater TP aphid acceptance. There were 2 clones per race, 5-11 replicates per clone and plant species. Mean ± SEM. Red = native host for MS aphids, blue = native host of TP aphids.

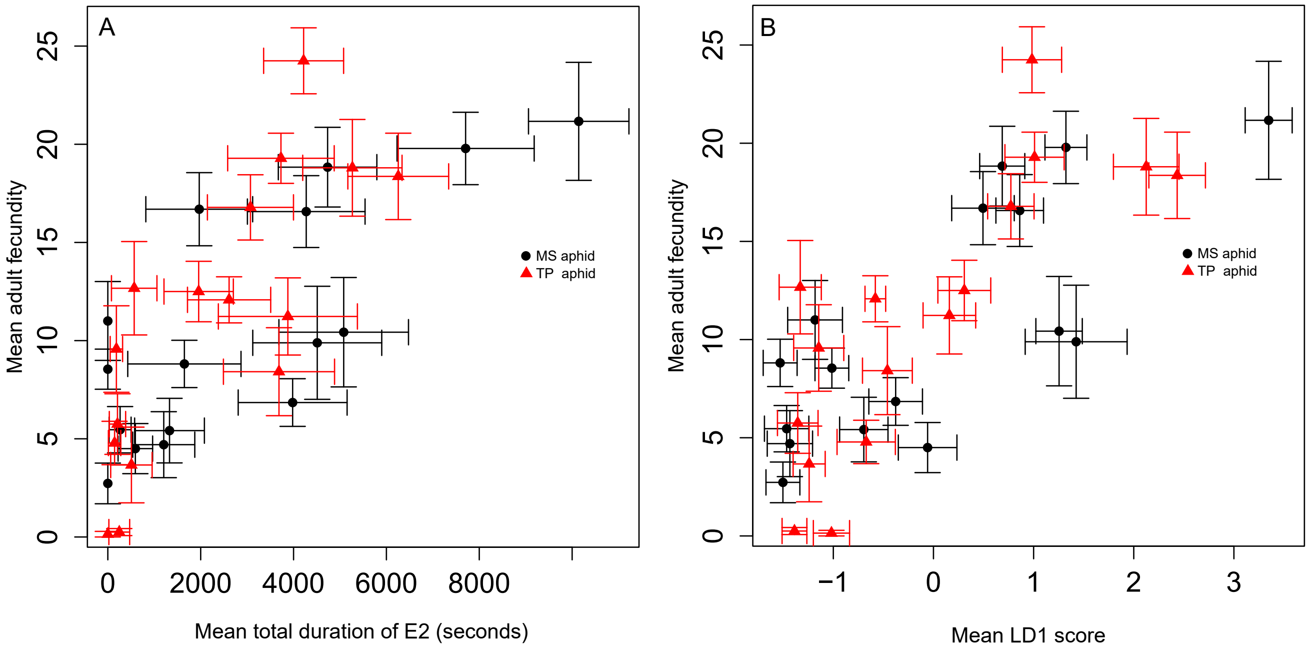

Figure S2. Correlation of mean aphid fecundity per plant species and aphid race with (A) total duration of E2 (r_S = *0.53, P*<0.001) and (B) LD1 discrimination profile (r_S = *0.78, P*<0.001). Mean ± SEM. Significance was tested with Spearman's rank correlation coefficient. Black - MS aphids, Red - TP aphids. DF= 17

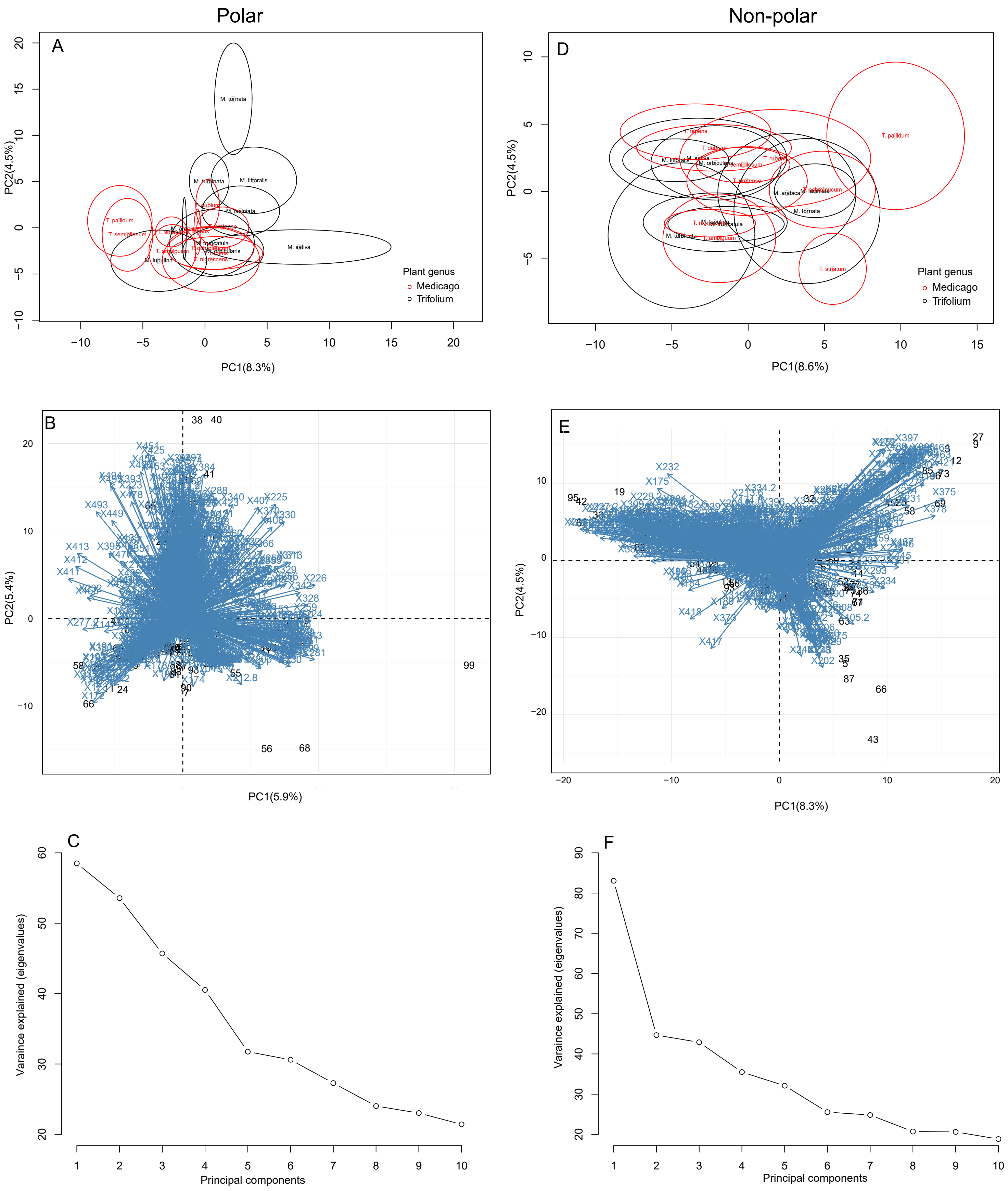

Figure S3. First two axes and loadings from PCA of (A-B) polar metabolomic data (=955 m/z bins) and (D-C) non-polar metabolomic data (=965 m/z bins). Points represent the mean scores and ellipses the standard errors for each of the plant species (A,D). Scree plots for (C) polar and (F) non-polar show the variance explained by the first 10 principle components. Both PCAs were performed with outlier data points removed (one *M. littoralis* plant in the polar data set and one *T. dubium* plant in the non-polar data set).

A) Polar data

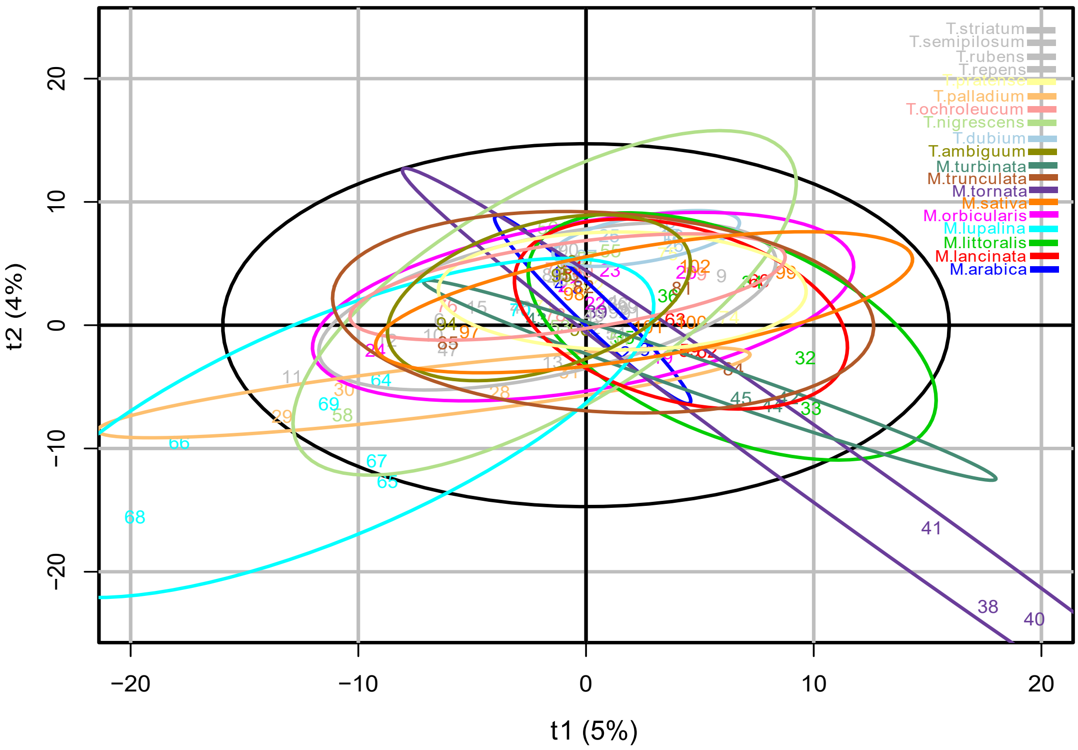

B) Non-polar data

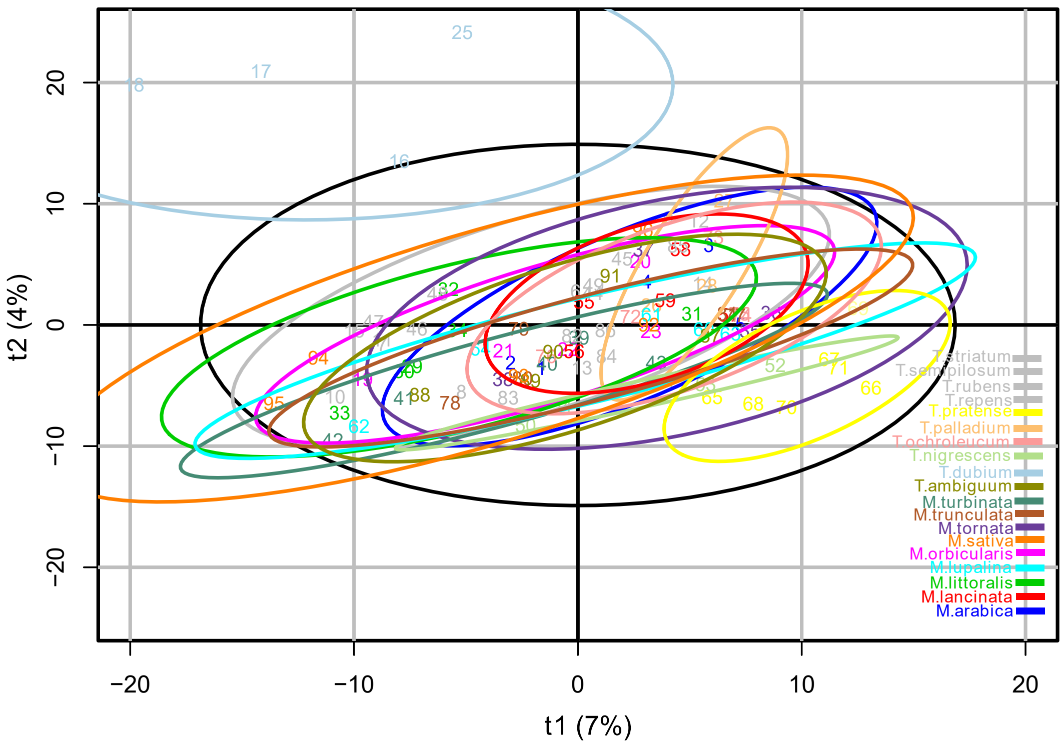

Figure S4. PLS plot of A) polar and B) non-polar metabolic data using plant species to classify groups. Both PLS analyses were performed with outlier data points removed (one *M. littoralis* plant in the polar data set and one *T. dubium* plant in the non-polar data set).

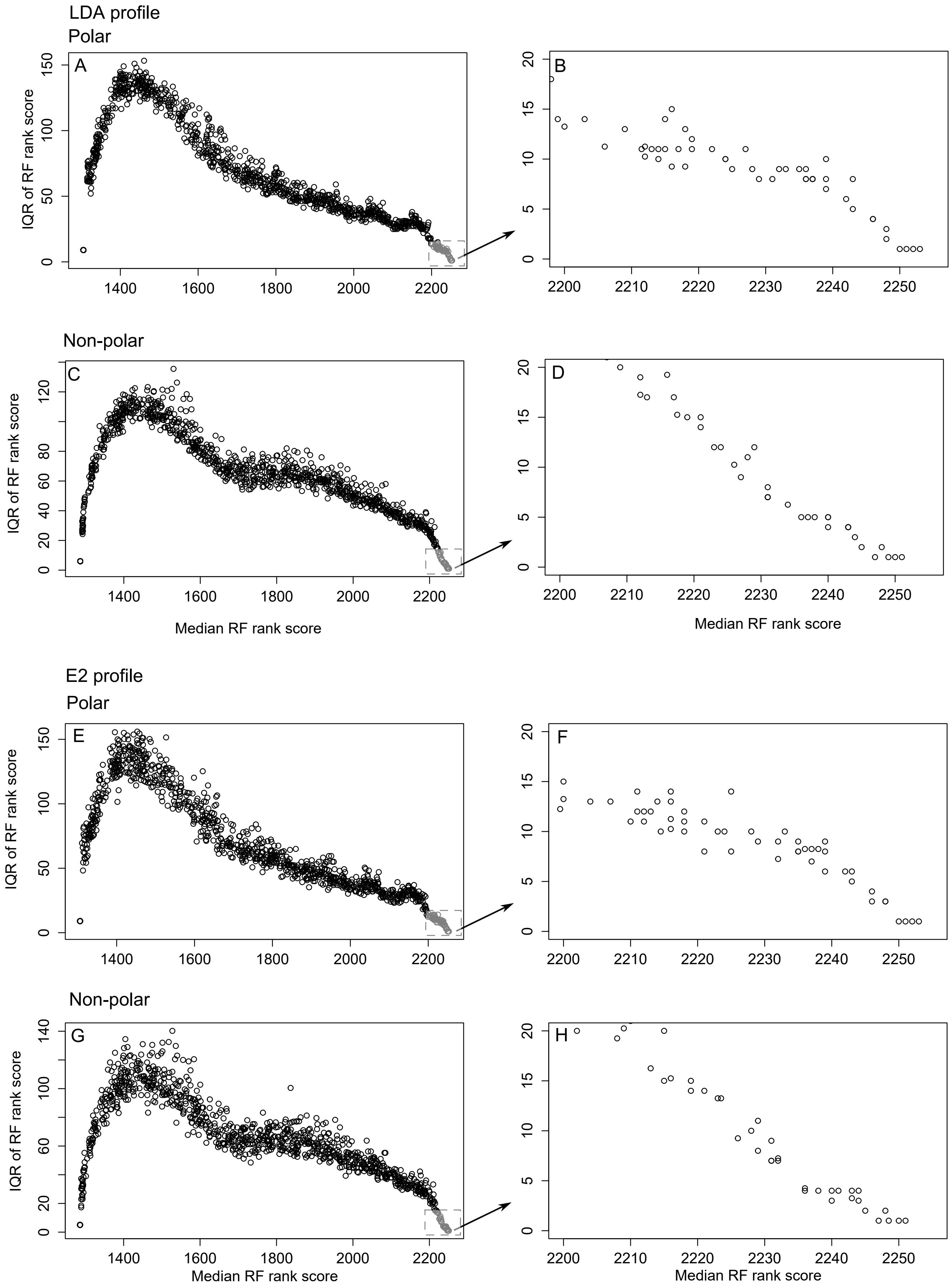

Figure S5. Summary of the RF model stability test comparing the interquartile rage (IQR) of 500 RF models run with jack-knifed E2 (E:D) and LDA (E:F) EPG profiles to the rank importance of m/z values in the original RF model. EPG profiles where jack-knifed by randomly removing one datum from each of the plant-aphid combinations before the E2 or LDA profiles were calculated.

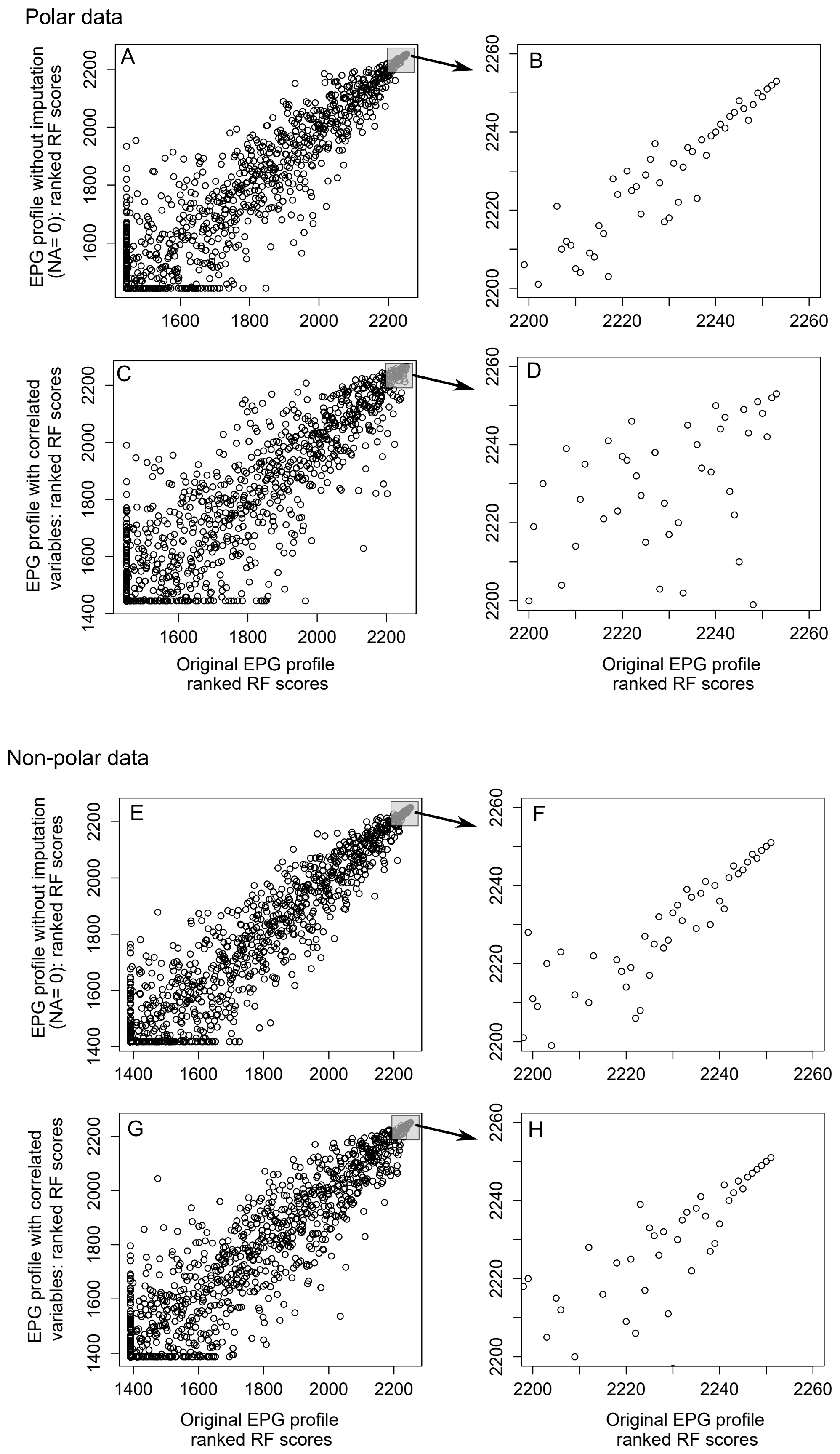

Figure S6. Comparison of original RF model results with the results of RF models that use EPG data for which (A:B, E:F) missing data points are treated as zeroes and (C:D,G:H) when the highly correlated waveforms are not removed . Models were tested using both (A:D) polar and (E:H) non-polar m/z value data. Figures on the left (A, D,E,F) show RF model results for all m/z values, while in figures on the right (B,D,F,H) only the most important m/z values are shown.

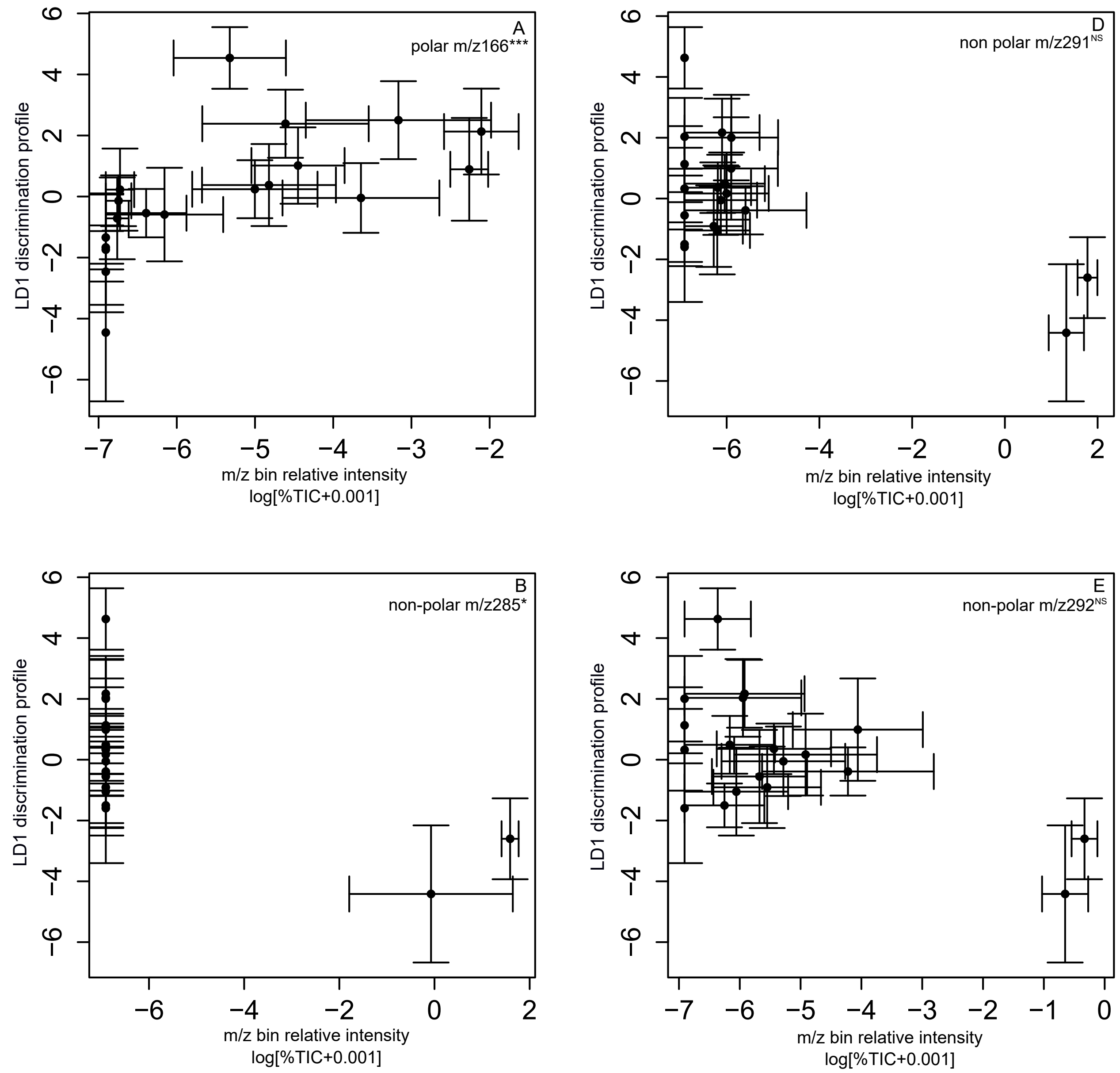

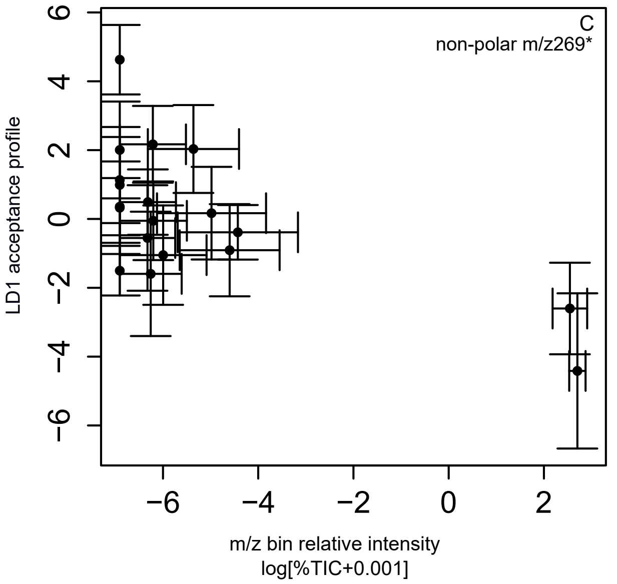

Figure S7. Relative intensity of m/z bins identified by RF models but not included in Figure 2 (main text), from polar or non-polar metabolomics profiles, in relation to LD1 discrimination scores. Mean ± SEM. Significance tested with Spearman's rank correlation with FDR correction. * $P< 0.05$, ** $P< 0.01$, *** $P< 0.001$.

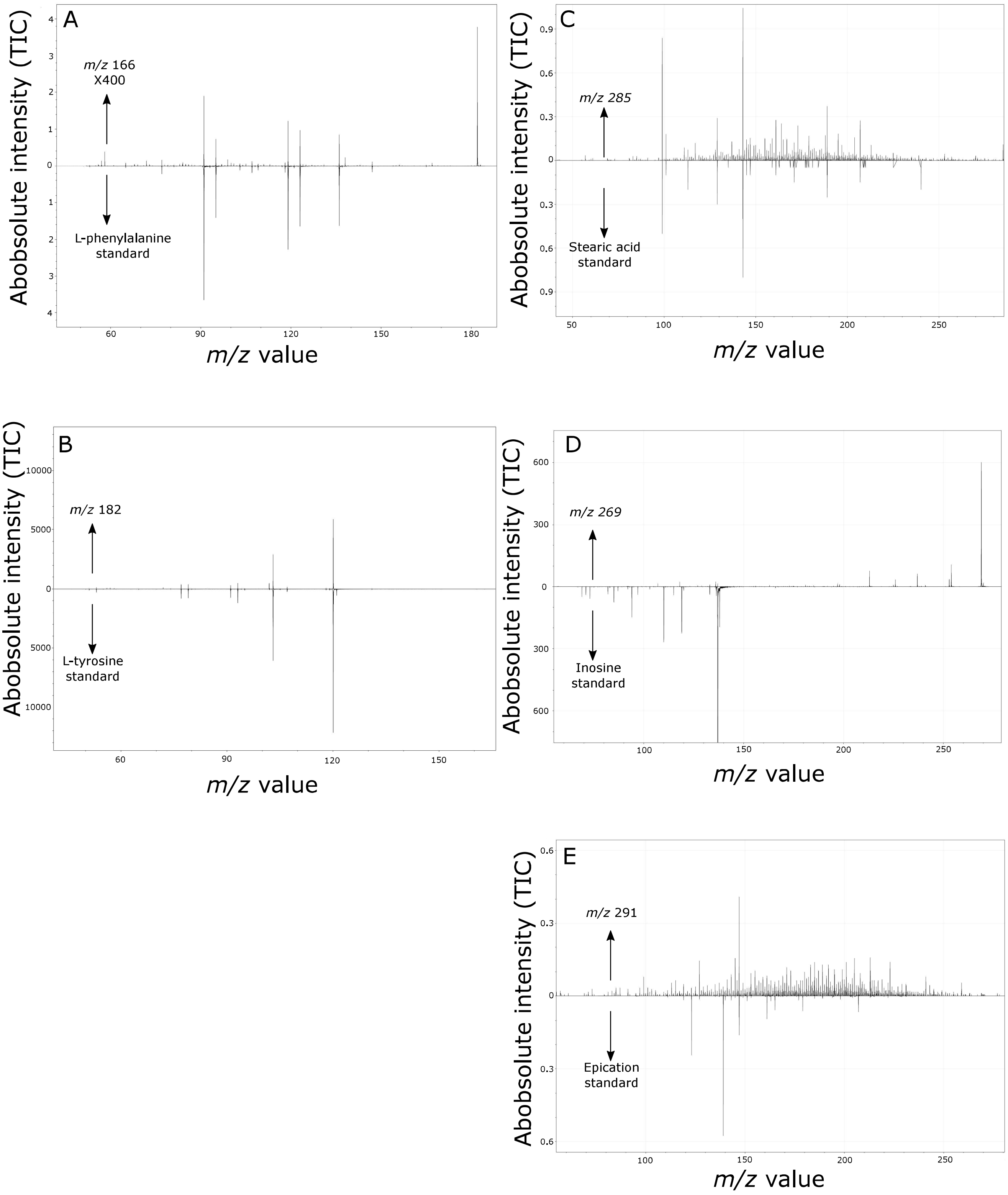

Figure S8. Tandem mass spectrometry plots of fragmentation patterns. (A) m/z bin 166 in polar samples against L-phenyalanine and (B) m/z bin 182 in polar samples against L-tyrosine, (C) m/z bin 166 in non-polar samples against Stearic acid, (D) m/z 269 in non polar samples against Inosine and (D) m/z 291 in non-polar samples against Epication. The unmatched peak at the far right of plot (B) is at m/z 182 so is likely to be the target compound which remained unfragmented within the more complex extract matrix.

Table S1: List of plant species used and their seed sources

Species	Source	Details of original source
M. arabica	IBERS, Aberystwyth University, Wales	Donated 1843, Denmark, University of Copenhagen Botanical Garden
T. rubens	IBERS, Aberystwyth University, Wales	Donated sample, 886466, ex England, Ardingly, International Plant Genetic Resources Institute, Seed Handling Unit. Collected Bovec, Tolmin, Slovenia.
T. semipilosum	IBERS, Aberystwyth University, Wales	Cv. Safari. Donated, 69, Ex Wales, Bangor University
T. dubium	IBERS, Aberystwyth University, Wales	Coll. Czechoslovakia 1992
M. orbicularis	IBERS, Aberystwyth University, Wales	Donated 1861, University of Copenhagen Botanical Garden
M. littoralis	IBERS, Aberystwyth University, Wales	Donated, 1119, ex Portugal, Coimbra, Jardim Botânico da Universidade Coimbra
M. tornata	IBERS, Aberystwyth University, Wales	Donated sample, 1869, University of Copenhagen Botanical Garden
M. turbinata	IBERS, Aberystwyth University, Wales	Dinated sample, 1871, ex Denmark, University of Copenhagen Botanical Garden
T. striatum	IBERS, Aberystwyth University, Wales	Donated, ex. France, Guyancout, INRA
T. nigrescens	IBERS, Aberystwyth University, Wales	Quinequeli. Donated ex Chile, Temuco, INIA, Estacion Experimentales Garillanca per Fernando Ortega
M. laciniata	University of Sheffield	Original plants taken from Kew Gardens, UK
M. lupulina	Emorsgate Gate	Origin: Norfolk . UK
T. pratense ssp. pratense	IBERS, Aberystwyth University, Wales	Cv. AberChianti. Ex Aa 4494. Diploid red clover bred for enhanced persistence under cutting and grazing.
T. ochroleucum	Chiltern Seeds©	Origin: Oxfordshire, UK
M. truncatula	IBERS, Aberystwyth University, Wales	Breeder line, 2005. Drought selection ex Af 1734 produced in compartment 3 of Venlo. (ABY-Af 1738-2005)
T. repens Var. small leaved	Emorsgate Gate	Origin: Amenity
T. ambiguum	IBERS, Aberystwyth University, Wales	Cv. Summit. Ex Australia, Canberra, CSIRO, Division of Plant Industry (ABY-Ah 1475-)
M. sativa ssp. sativa	IBERS, Aberystwyth University, Wales	Cv. Sabilit. Ex AF7 (AF1)

Table S2. EPG waveform measurements used in the PCA and LDA analyses (after removal of variables with correlations >0.80; all durations in seconds; measurements are defined in Sarria et al.[26])

No.	Waveform	No.	Waveform
1	Time to 1st probe from start of EPG	37	Total duration of np during the 5th hour
2	Number of probes to the 1st E1	38	Total duration of np during the 6th hour
3	Number of F	39	Number of F during the 1st hour
4	Duration of 1st probe	40	Number of F during the 2nd hour
5	Duration of 2nd probe	41	Number of F during the 3rd hour
6	Duration of the shortest C wave before E1	42	Number of F during the 4th hour
7	Duration of the second non-probe period	43	Number of F during the 5th hour
8	Total duration of F	44	Number of F during the 6th hour
9	Mean duration of F	45	Total duration of F during the 1st hour
10	Number of G	46	Total duration of F during the 2nd hour
11	Duration of G	47	Total duration of F during the 3rd hour
12	Number of probes after 1st E	48	Total duration of F during the 4th hour
13	Number of E1	49	Total duration of F during the 5th hour
14	Number of E1 longer than 10 minutes followed by E2	50	Total duration of F during the 6th hour
15	Number of single E1	51	Number of probes during the 1st hour
16	Duration of 1st E	52	Number of probes during the 2nd hour
17	Duration of the E1 followed by first sustained E2 longer than 10 min	53	Number of probes during the 3rd hour
18	Potential E2 index	54	Number of probes during the 4th hour
19	Total duration of E	55	Number of probes during the 5th hour
20	Total duration of E1	56	Number of probes during the 6th hour
21	Total duration of single E1	57	Time from the beginning of E1 to the end of the EPG record
22	Number of probes	58	Time from the beginning of E2 to the end of the EPG record
23	Number of C	59	Duration of np just after the probe of the first sustained E2
24	Number of E1e	60	% of time probing spent in C
25	Total duration of C		
26	Total duration of E1e		
27	Total probing time		
28	Mean duration of np		
29	Mean duration of C		
30	Time to from start of EPG 1st sustained E2 longer than 10 minutes		
31	Time from the beginning of that probe to 1st sustained E2 longer than 10 minutes		
32	Time from the beginning of that probe to 1st E2y		
33	Total duration of np during the 1st hour		
34	Total duration of np during the 2nd hour		
35	Total duration of np during the 3rd hour		
36	Total duration of np during the 4th hour		

Table S3. Top 20 m/z bins based on their median rank RF importance (measured as *Mean Decrease Gini*).

**Nonpolar, E2 profile MS aphid – TP aphid**		**Polar, E2 profile MS aphid – TP aphid**		**Nonpolar, E2 profile MS aphid + TP aphid**		**Polar, E2 profile t MS aphid + TP aphid**		**Non polar, lda profile MS aphid – TP aphid**		**Polar, lda profile MS aphid - TP aphid**		**Nonpolar, lda profile MS aphid + TP aphid**		**Polar, lda profile MS aphid + TP aphid**	
**m/z value**	median rank	**m/z value**	median rank	**m/z value**	median rank	**m/z value**	median rank	**m/z value**	median rank	**m/z value**	median rank	**m/z value**	median rank	**m/z value**	median rank
**166**	2245	**182**	2252	**166**	2236	**250**	2240	**182**	2250	**182**	2253	**137**	2230	**137**	2252
**182**	2245	**183**	2249	**182**	2232	**176**	2237.5	**166**	2249	**183**	2252	**189**	2230	**250**	2244
**292**	2229	**331**	2247	**250**	2225	**331**	2234.5	**269**	2249	**309**	2247	**250**	2225	**417**	2234
**269**	2226	**484**	2247	**331.2**	2210	**182**	2228	**292**	2248	**166**	2245	**166**	2224	**184**	2232
**250**	2221	**309**	2246	**234**	2208	**137**	2226	**291**	2247	**345**	2245	**341.2**	2224	**176**	2231
**291**	2217	**137**	2245	**266**	2205	**138**	2222	**182.6**	2246	**363**	2244	**297.2**	2221.5	**345**	2231
**138**	2209	**363**	2242	**471.2**	2205	**229.2**	2222	**285**	2244	**361**	2242	**234**	2221	**477**	2231
**212**	2202	**385**	2241	**292**	2201	**301**	2220	**184.6**	2242	**385**	2242	**445**	2219	**435**	2226
**331.2**	2201.5	**250**	2236	**138**	2198	**184**	2218	**177**	2238	**145.2**	2240	**191**	2217.5	**266**	2224
**471.2**	2199.5	**196**	2235	**212**	2198	**487**	2218	**138.6**	2234	**331**	2240	**204.6**	2217	**325**	2222
**234**	2197	**357.2**	2235	**445**	2192	**417**	2217	**307**	2234	**341**	2240	**352**	2217	**379**	2222
**160**	2196	**176**	2234	**160**	2191	**435**	2217	**339.2**	2234	**325**	2238	**195**	2214	**196**	2221
**339.2**	2194.5	**325**	2234	**269**	2190	**266**	2215	**167**	2227	**347**	2235	**395**	2211	**229.2**	2221
**395**	2194	**166**	2232	**373**	2188	**345**	2211.5	**308**	2225	**484**	2232	**386.2**	2210	**189.2**	2220
**184.6**	2192.5	**344**	2232	**477**	2188	**347**	2211	**250**	2221	**196**	2230	**361**	2209	**331**	2220
**167**	2191	**136**	2231	**220.2**	2187	**183**	2210.5	**240.2**	2220	**357.2**	2230	**192**	2202	**363**	2218
**477**	2191	**348**	2231	**337.2**	2185	**287**	2210.5	**144.6**	2217	**176**	2229	**425.2**	2201	**119**	2216
**373**	2190	**292**	2229	**395**	2180	**189.2**	2205	**206**	2217	**271**	2226	**266**	2200.5	**418**	2216
**266**	2189	**347**	2222	**232**	2179	**222**	2201.5	**212**	2213	**401**	2225	**410.2**	2199.5	**487**	2216
**337.2**	2184.5	**417**	2219	**192**	2178	**353**	2200.5	**180**	2212	**324**	2224	**454.2**	2199.5	**202**	2214

Table S4: Change in order of importance of the top 8 m/z values from the original RF model when outliers were removed from the metabolomics data sets.

Non-polar, E2 profile MS aphid – TP aphid		Polar, E2 profile MS aphid – TP aphid		Non-polar, E2 profile MS aphid + TP aphid		Polar, E2 profile MS aphid + TP aphid		Non polar, LD1 profile MS aphid – TP aphid		Polar, LD1 profile MS aphid - TP aphid		Non-polar, LD1 profile MS aphid + TP aphid		Polar, LD1 profile MS aphid + TP aphid	
**m/z value**	order change	**m/z value**	order change	**m/z value**	change in order	**m/z value**	order change	**m/z value**	order change	**m/z value**	order change	**m/z value**	order change	**m/z value**	order change
**166**	0	**182**	0	**166**	0	**250**	0	**182**	0	**182**	0	**137**	-2	**137**	0
**182**	0	**183**	-4	**182**	0	**176**	0	**166**	0	**183**	0	**189**	0	**250**	0
**292**	-2	**331**	-1	**250**	0	**331**	-1	**269**	0	**309**	-2	**250**	-2	**417**	-4
**269**	+1	**484**	-196	**331.2**	-2	**182**	-1	**292**	0	**166**	+1	**166**	0	**184**	-1
**250**	+1	**309**	-3	**234**	-3	**137**	+2	**291**	0	**345**	+1	**341.2**	-5	**176**	+1
**291**	-7	**137**	-4	**266**	-1	**138**	0	**182.6**	0	**363**	0	**297.2**	-2	**345**	-2
**138**	-1	**363**	-11	**471.2**	-2	**229.2**	0	**285**	0	**361**	0	**234**	+1	**477**	+4
**212**	-2	**385**	-134	**292**	+3	**301**	-4	**184.6**	-1	**385**	-2	**445**	-3	**435**	-3
